# Unveiling the Potential of Haloalkenes as Electron Density Acceptors

**DOI:** 10.1021/acs.cgd.4c00538

**Published:** 2024-06-24

**Authors:** Juan D. Velasquez, Noushin Keshtkar, Víctor Polo, Julen Munárriz, Jorge Echeverría

**Affiliations:** † Instituto de Síntesis Química y Catalisis Homogénea (ISQCH) and Departmento de Química Inorgánica, Facultad de Ciencias, Universidad de Zaragoza, Pedro Cerbuna 12, 50009 Zaragoza, Spain; ‡ Departmento de Química Física, Facultad de Ciencias, Universidad de Zaragoza, Pedro Cerbuna 12, 50009 Zaragoza, Spain; § Instituto de Biocomputación y Física de Sistemas Complejos (BIFI), Universidad de Zaragoza, 50009 Zaragoza, Spain

## Abstract

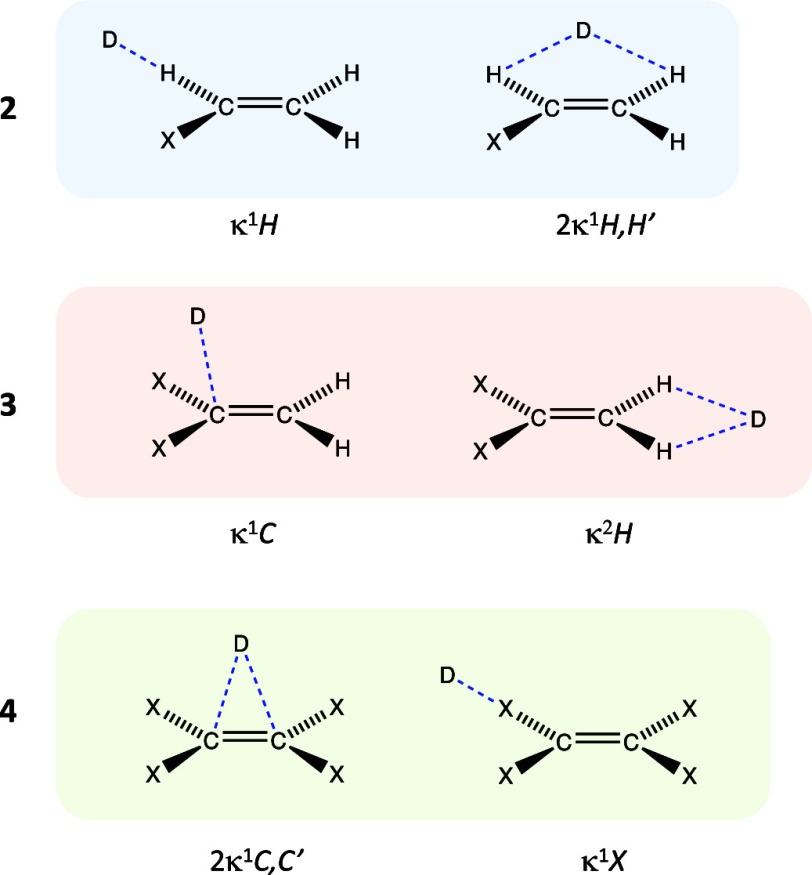

We report herein, by means of structural and computational analyses, a comprehensive study of the capability of differently substituted haloalkenes to behave as electron density acceptors in noncovalent interactions. The nature of these interactions between haloalkenes and Lewis bases highly depends on the number and nature of the halogen atoms bound to the carbon–carbon double bond. When hydrogen bonds, which generally dominate for mono- and dihaloalkenes, cannot be formed, we observe the establishment of attractive interactions in which an sp^2^ carbon atom, belonging to an acyclic C=C double bond, plays the role of the Lewis acid via its π* antibonding orbital.

## Abstract 2

By means of a computational study, we have evaluated the capability of halogenated ethene systems to engage in noncovalent interactions as the electron-deficient species, involving different interaction modes depending on the halogen atom present and the nature of the Lewis base.

## Introduction

Noncovalent interactions play a determining role in the final conformation and stability of both organic and inorganic molecules and in dictating the crystal structures of molecular systems.^1,2^ More specifically, interactions between a lone pair and the electron deficient region of a π system have attracted much interest in recent years.^3^ Probably, the most paradigmatic case of lone pair-π interaction is that between two carbonyl groups. The overlap between the O-based lone pair of one carbonyl and the π* antibonding orbital of the other leads to a small energy release (∼0.3 kcal/mol) that is yet responsible for the stabilization of specific conformations of lactones,^4^ peptoids,^5^ organic compounds,^6,7^ and transition metal complexes.^8,9^ Recently, analogous interactions have been found with cyano,^10^ thiocyano,^11^ and isocyano.^12^ On the other hand, interactions involving aromatic systems acting as the Lewis acid have also been investigated. However, it must not be forgotten that many of the so-called lone pair–π or anion–π interactions with electron-deficient rings actually involve σ_C-R_ orbitals (e.g., σ_C-F_ in C_6_F_6_) as the charge transfer acceptors rather than π*_C=C_ orbitals of the aromatic system.^13^ Regarding electron-rich aromatic systems, Das and co-workers recently reported spectroscopic evidence for an n → π* interaction between a carbonyl group (Lewis base) and the phenyl ring (Lewis acid) of phenyl formate.^14^ In that case, the acceptor is a π*_C=C_ orbital, and the stabilizing interaction is probably due to the combination of charge transfer and dispersion that is able to overcome Coulombic and Pauli repulsion. Although the nature of the n → π* interaction is eminently orbital, electrostatics usually plays a significant role in the system stabilization, which makes it difficult to make a clear distinction between n → π* and π–hole interactions.

Aliphatic systems do not usually create π–holes because the presence of the two carbon atoms lead to π molecular orbitals with lobes of similar sizes with little or no charge displacement along the bond. On the other hand, in carbonyl groups, the electron density is attracted by the oxygen atom, leading to a π_C=O_ bonding orbital mostly located on the oxygen and a π*_C=O_ antibonding mostly located on the carbon atom, the latter being the origin of the π-hole. At this point, we wonder whether carbon–carbon double bonds can engage in noncovalent interactions as the electron-deficient species. In order to do so, the electron density along the C=C bond must be reorganized to resemble that of a carbonyl, for instance, by substitution effects. A few years ago, Kozuch showed that a π–hole appears in F_2_C=CH_2_ in a similar fashion to what happens in H_2_C=O.^13^ Furthermore, it is well-known that carbon–carbon double bonds activated by electron-withdrawing groups can be attacked by different nucleophilic species giving place to a variety of products.^15^ In the light of this, we undertake herein a computational and structural analysis of noncovalent interactions involving sp^2^ carbon atoms in haloalkenes as the electron-poor partner. Accordingly, we will study mono-, di-, and tetrahalogenated ethene molecules by means of DFT calculations, paying special attention to the different interaction modes that these systems can establish with electron-rich species.

## Results and Discussion

**Scheme 1:**
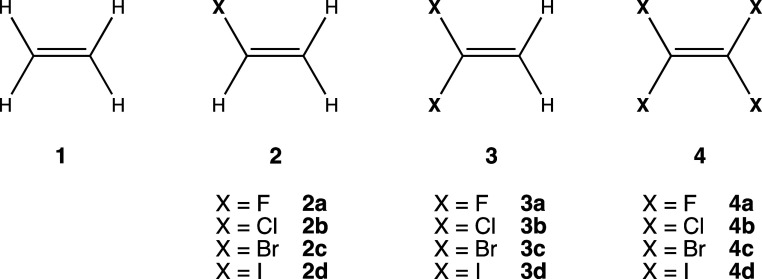
Alkenes Investigated in This Work

First, we analyze the adducts established between two different Lewis bases, both neutral (acetone) and charged (chloride), and the differently substituted C=C bonds as the Lewis acids (Scheme 1). For the analysis of the interatomic distances, we will make use of the newly defined penetration index (*p*_AB_), a parameter that evaluates the interpenetration of the van der Waals crusts of two atoms, A and B, allowing comparison regardless of their sizes (see the section Computational Details for further information on the use of penetration indices).^16^ The use of this parameter is particularly useful for comparing distances between atoms of different nature, and it has been recently used to unify and rationalize the dimerization processes of halocomplexes involving group 11 and 12 transition metals.^17^

**Figure 1:**
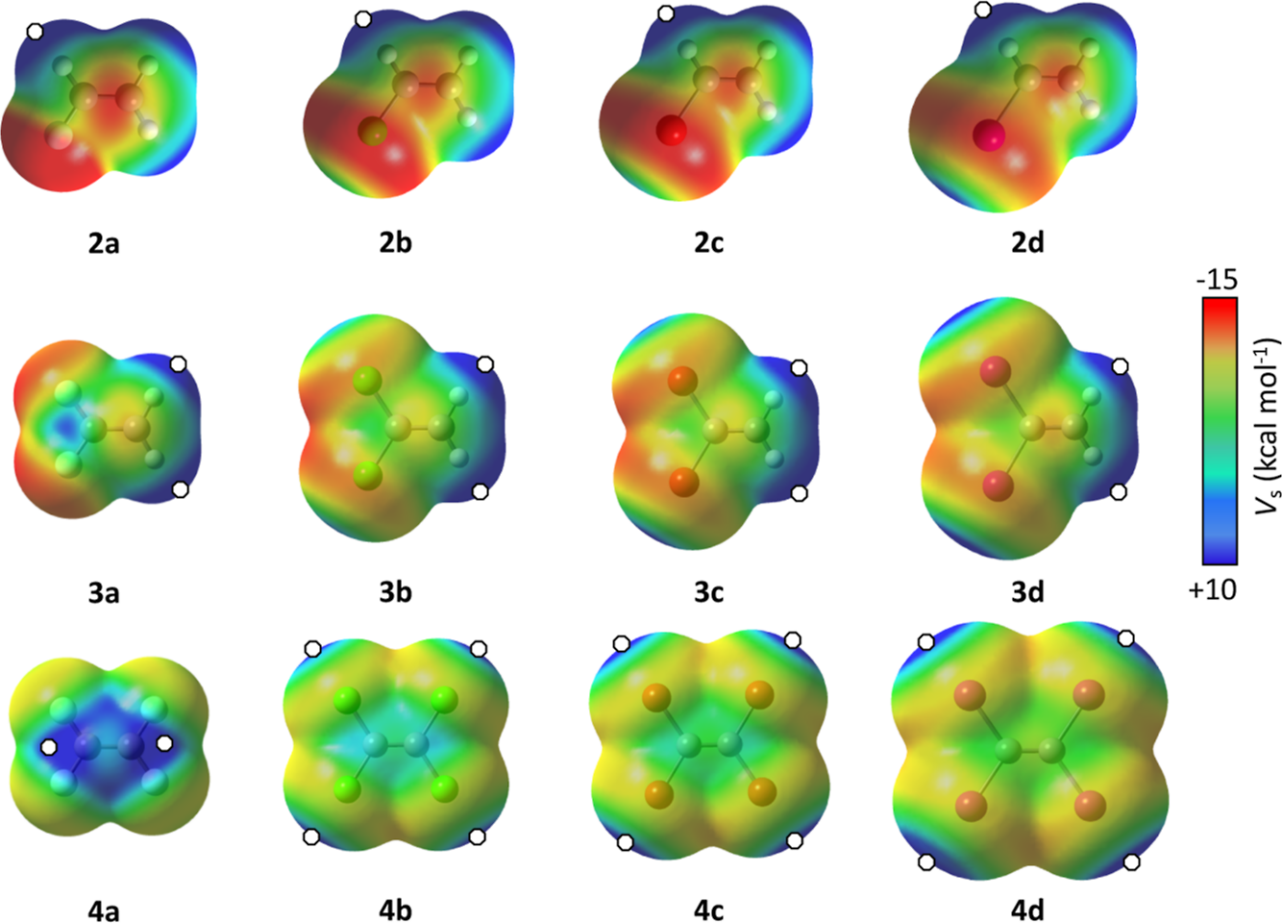
MEP maps of compounds **2**–**4** with white points indicating the position of maximum electrostatic potential values (*V*_s,max_).

Before optimizing the adducts, one can try to forecast the most favorable interaction geometries by identifying the most electrophilic regions of the different halogenated ethenes. Accordingly, we have plotted the molecular electrostatic potential (MEP) maps of **2–4** on the corresponding van der Waals isosurfaces (Figure 1). In the case of **2**, the most positive value of the MEP (*V*_s,max_) is located at the H atom closer to the halogen in all four alkenes. As for **3**, again, the *V*_s,max_ corresponds to the H atoms, although there is a marked region of electron density depletion around the halogenated carbon atom in **3a**. Finally, for fully halogenated alkenes **4**, the heavier halogen atoms show the characteristic σ–holes in **4b**-**c**, while for **4a**, there appear to be two π–holes associated with the two carbon atoms.

**Figure 2:**
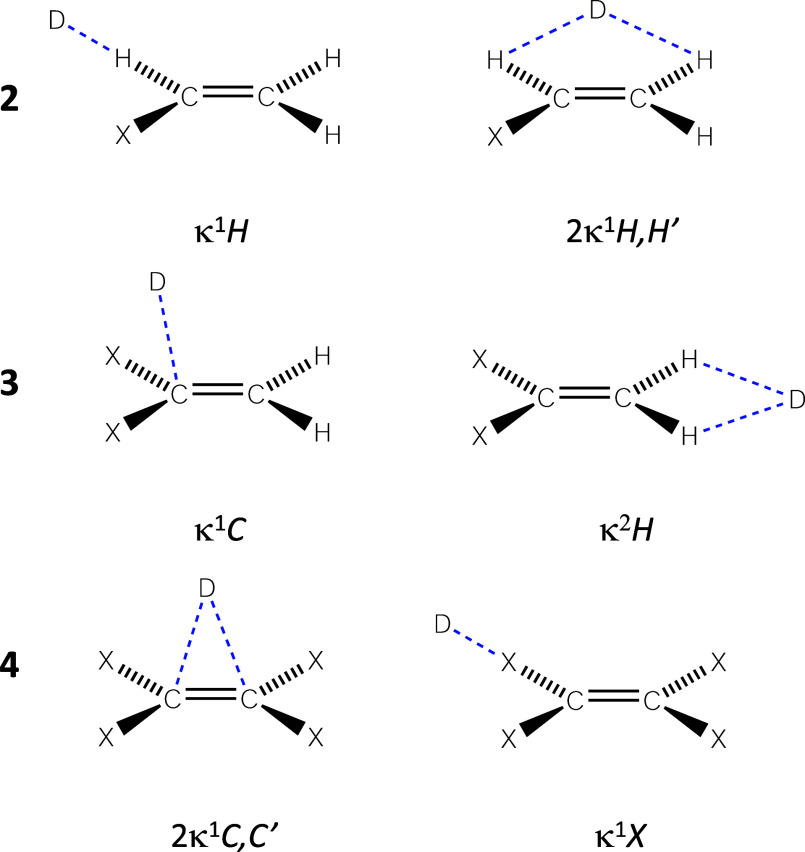
Main interaction modes for adducts formed by a haloalkane (**2–4**) and an electron density donor system (D).

Following, we have optimized the adducts formed between **2** and **4** and two different Lewis bases (D), one charged (chloride) and the other neutral (acetone). The results are summarized in Tables 1 and 2, respectively, and the main interaction geometries found are depicted in Figure 2. In general, all calculated interactions strengthen as we increase the size of the halogen atom, even for those in which the halogen atom is not directly involved. In the case of chloride as the Lewis base, for **2** and **3**, interaction topologies are dominated by hydrogen bonds (2κ^1^H,H′ and κ^2^H conformations for **2** and **3**, respectively) as expected in the light of the calculated MEP maps. For adducts with **4**, there are two possible outcomes. On one side, the Lewis base can establish a halogen bond with the corresponding halogen atom to give a κ^1^X conformation, and on the other side, the Lewis base can be perpendicular to the C=C bond to interact with the two carbon atoms in a 2κ^1^C,C′ conformation. Halogen bonds κ^1^X are more stabilizing than 2κ^1^C,C′ interactions in all cases, but the difference in energy is significantly larger for **4d**, which can be attributed to the capability of iodine to form deeper σ–holes. Note that since fluorine does not generate σ–holes, the adduct with **4a** was only isolated in a 2κ^1^C,C′ conformation.

**Table 1:** Main Geometrical Parameters and Interaction Energies for the Fully Optimized Adducts Formed by Chloride and Haloalkanes **1–4** Calculated at the M06-2X/def2-TZVPD Level

alkene	interaction mode	*p*_Cl···C_ (%)	ang_Cl···C=C_ (°)	Δ*E*_int_ (kcal/mol)
**1**	κ^2^H			–5.00
**2a**	2κ^1^H,H′			–10.63
**2b**	2κ^1^H,H′			–11.81
**2c**	2κ^1^H,H′			–12.46
**2d**	2κ^1^H,H′			–12.60
**3a**	κ^2^H			–9.79
**3b**	κ^2^H			–11.22
**3c**	κ^2^H			–11.85
**3d**	κ^2^H			–12.09
**4a**	2κ^1^C,C′	26.6	88.2	–8.35
**4b**	2κ^1^C,C′	19.0	78.1	–8.32
	κ^1^X			–10.01
**4c**	2κ^1^C,C′	19.0	78.2	–8.85
	κ^1^X			–16.06
**4d**	2κ^1^C,C′	17.9	78.3	–9.90
	κ^1^X			–28.39

**Table 2:** Main Geometrical Parameters and Interaction Energies for the Fully Optimized Adducts Formed by Acetone and Alkanes **1–4** Calculated at the M06-2X/def2-TZVPD Level

alkene	interaction mode	*p*_O···C_ (%)	ang_O···C=C_ (°)	Δ*E*_int_ (kcal/mol)
**1**	κ^2^H			–3.17
**2a**	κ^1^H			–3.96
**2b**	κ^1^H			–4.35
**2c**	κ^1^H			–4.56
**2d**	κ^1^H			–4.62
**3a**	κ^1^C	15.5	106.4	–2.55
**3b**	κ^1^C	9.2	93.2	–3.09
	κ^2^H			–1.99
**3c**	κ^1^C	8.2	89.7	–3.24
	κ^2^H			–2.08
**3d**	κ^1^C	7.1	88.1	–3.05
	κ^2^H			–1.98
**4a**	κ^1^C/2κ^1^C,C′	23.4	100.7, 56.5	–3.06
**4b**	2κ^1^C,C′	15.4	75.8, 78.5	–3.98
	κ^1^X			–2.62
**4c**	2κ^1^C,C′	16.0	74.5, 80.0	–4.21
	κ^1^X			–3.51
**4d**	2κ^1^C,C′	13.8	74.0, 81.0	–4.45
	κ^1^X			–5.16

If we look now at the adducts with a neutral donor system (namely, acetone), some differences can be found. For **2**, κ^1^H conformation is preferred over 2κ^1^H,H′ because of the possibility of hydrogen bonding between the covalently bonded halogen atom and one of the methyl groups from the ketone. For molecules **3**, κ^1^C topology is more stable than hydrogen bonded adducts with κ^2^H topology. Note that we could not find a κ^2^H conformation for **3a**. It is also worth noticing that hydrogen bonds are stronger in ethene (**1**) than in halosubstituted analogues **3b**–**d**. Finally, for **4**, the differences in energy between κ^1^X and 2κ^1^C,C′ are smaller than in the case in which the Lewis base is chloride. In fact, for **4b** and **4c**, the halogen bond is less stabilizing than the lone pair–π interaction associated with a 2κ^1^C,C′ topology.

**Figure 3:**
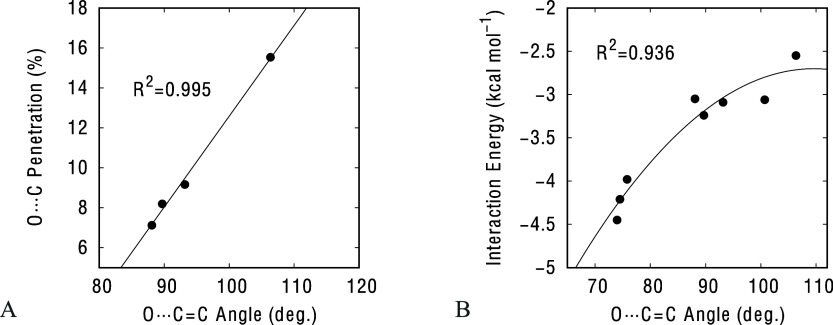
Dependence of the (A) O···C penetration index with the O···C=C attack angle for acetone···**3** adducts with κ^1^C interaction mode and (B) of the O···C=C attack angle with the interaction energy for acetone···**3**–**4** adducts with κ^1^C and 2κ^1^C,C′ interaction modes.

If we narrow our focus on the lone pair–π interactions involving the C=C framework, we observe that the calculated interaction energies range from −2.5 to −10 kcal/mol, while the Cl···C and O···C penetration indices are between 17.9 and 26.6% for chloride and between 13.8 and 23.4% for acetone, respectively. It is also interesting to observe the correlation between the O···C penetration index and the O···C=C attack angle in κ^1^C adducts: the shorter the distance, the closer the angle to 107° (Figure 3A), which is the typical value associated with the Burgi–Dunitz trajectory.^18−20^ This behavior has been previously observed in other lone pair–π interactions involving carbonyl groups^8^ or aromatic rings.^6^ Remarkably, we also found a nice correlation between this O···C=C angle and the interaction energy for all systems interacting with acetone in κ^1^C and 2κ^1^C,C′ interaction modes (Figure 3B).

**Figure 4:**
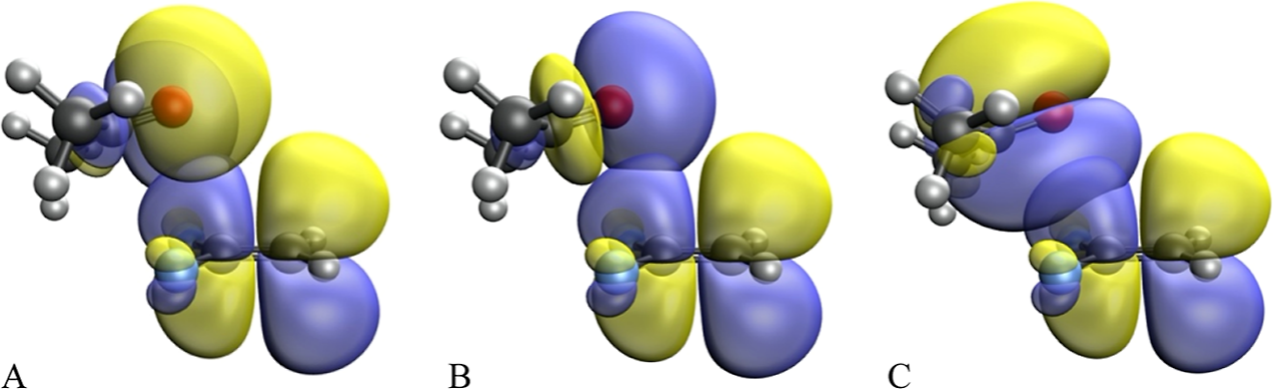
Natural bond orbitals involved in the n_O_ → π*_C=C_ charge transfer processes for the acetone-**3a** adduct with κ^1^C interaction mode from n_O_ donor orbitals with (A) sp and (B) p characters and (C) in the π_C=O_ → π*_C=C_ charge transfer process.

We have run natural bond orbital (NBO) calculations in order to unveil any possible charge transfer process between the Lewis base and the haloalkene (full results can be found in the Supporting Information). If we look at adducts with κ^1^C and 2κ^1^C,C′ interaction modes with acetone, while the acceptor orbital is always the π* antibonding orbital of the C=C bond, there appear three different donors; on one side, the two lone pairs of the oxygen atom (Figure 4A,B) and, on the other side, the π bonding orbital of the carbonyl group (Figure 4C). The corresponding second-order perturbation energies range between 0.05 and 0.40 kcal/mol. For the case in which the Lewis base is chloride, the energies are significantly larger (0.20–1.45 kcal/mol). Moreover, the short distance between chloride and the haloalkane allows for a charge transfer from the chloride lone pair into the σ-antibonding of the C–F bond.

We next check if there are experimental examples of short Lewis base···C=C contacts that can be constitutive of stabilizing interactions. In the case of haloalkanes of type **2**, we did not find any experimental examples interacting with donor systems. However, if we allow the substituents of the nonhalogenated carbon to be any atom, only 8 of the 55 structures found with intermolecular D···C=C contacts shorter than the vdW radii sum do not display accompanying D···H–C=C hydrogen bonds. This is in excellent agreement with the previous computational analysis of type **2** models that predicted 2κ^1^H,H′ and κ^2^H to be the most favorable conformations.

**Figure 5:**
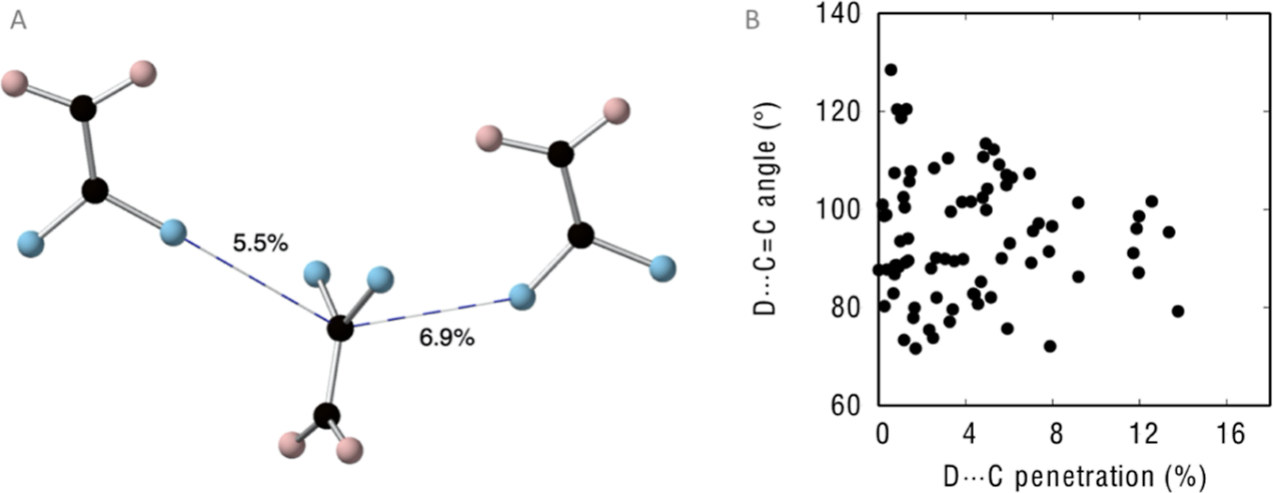
(A) Short F···C = C contacts along with the corresponding penetration indices in the crystal structure of 1,1-difluoroethene. (B) Dependence of the D···C (D = O, S, N, P, F, Cl, Br, and I) penetration with the D···C=C angle for contacts shorter than the sum of the vdW radii in dihaloethene derivatives as found in the CSD.

Regarding dihaloalkanes of type **3**, we have found the crystal structure of 1,1-difluoroethene (**3a**) reported by Lentz and co-workers as the only example.^21^ In such crystal structure, there are no F···H hydrogen bonds, and the molecules are held together by two F···C=C contacts in a κ^1^C interaction mode with p_F···C_ values of 5.5 and 6.9% and associated F···C=C angles of 109.2 and 107.3°, respectively (Figure 5A). It is worth noting that our calculated angle for **3a** is 106.4°, very close to the experimental values.

We have loosened our search criteria trying to find some directionality in the interactions established by dihaloalkanes of type **3** with electron density donors. Accordingly, we have allowed the two hydrogens in the F_2_C=CH_2_ molecule to be any atom (except halogens). Then, a search in the Cambridge structural database (CSD) for D···C=C contacts shorter than the sum of the van der Waals radii yields 78 structures, which show a geometrical preference: the shorter the D···C contact, the closer the D···C=C angle to 90–100° (Figure 5B), relatively close to the Bürgi–Dunitz angle^20^ (∼107°) previously found for other lone pair–π interactions.^8,22^

Finally, we found 5 crystal structures with tetrahaloalkanes (1 with F, 1 with Cl, and 3 with I) of type **4** displaying short D···C=C contacts. For X = F and X = Cl, the donor atom interacts with the two carbon atoms of the double bond with similar penetration indices (Δp_D···C_ < 2.5%), which can be rationalized in terms of 2κ^1^C,C′ interaction topology. One of the three structures with X = I also presents such an arrangement with D···C penetrations of 14.6 and 10.4%, respectively. In the other two cases, the adduct geometry resembles a κ^1^C interaction rather than a 2κ^1^C,C′ one.

## Conclusions

We have carried out a computational study on the capability of halogenated ethene systems to engage in noncovalent interactions as the electron-deficient species. While monohalogenated ethene molecules prefer to establish hydrogen bonds with Lewis bases, di- and tetrahalogenated ethenes can form lone pair–π interactions via their C=C frameworks. For these interactions, an attack angle close to 107° seems to maximize the interaction strength, which confers these lone pair–π contacts some degree of directionality. Accordingly, the analyzed interactions seem to be electrostatically driven but without ruling out some degree of orbital stabilization. On the other hand, the presence of a small and charged species as the Lewis base favor the formation of halogen bonds with the covalently bonded halogen atoms in the case of tetrahalogenated ethene molecules. The relatively large calculated interaction energies, between −2.5 and −10.0 kcal/mol, point toward a possible use of these interactions in crystal engineering, especially if hydrogen bonds are absent to minimize other competing interactions such as hydrogen bonds.

## Computational Details

Structural searches were carried out in the CSD, version 5.41 (November 2019) with 3 updates.^23^ Crystal structures with 3D coordinates defined, nondisordered, with no errors, not polymeric and with *R* lower than 0.1 were considered. For the analysis of interatomic distances, we used the recently proposed penetration index. The penetration index *p*_AB_ is a parameter that indicates the degree of interpenetration of the van der Waals crusts of atoms A and B from 0 (canonical vdW contact) to 100% (canonical bond distance) and is defined as *p*_AB_ = 100·(*v*_A_ + *v*_B_ – *d*_AB_)/(*v*_A_ + *v*_B_ – *r*_A_ – *r*_B_), where *v* is the van der Waals radius and *r* the covalent radius of a given atom. For computing *p,* we used standard sets of van der Waals^24^ and covalent^25^ radii. Further details on the use of penetration indexes and their applications can be found in a recent publication.^26^

DFT calculations were performed with the M06-2X functional and the def2-TZVPD basis sets^27^ for all atoms. We chose the M06-2X functional because it has shown reasonably good performance to deal with noncovalent interactions in previous benchmark reports.^28,29^ All adducts were fully optimized and characterized as true minima of the corresponding potential energy surfaces by diagonalization of the Hessian matrix. Interaction energies were calculated via the supermolecule approach and corrected for the BSSE by means of the counterpoise method.^30^ All electronic structure calculations were carried out with Gaussian16.^31^ MEP maps were built on the corresponding *s* = 0.001 au isosurfaces.

## Supporting Information Available

Complete NBO results and Cartesian coordinates of all optimized systems (PDF)
