# Supplementary material for: Unveiling the Potential of Haloalkenes as Electron Density Acceptors
Source: Cryst Growth Des. 2024 Jun 24;24(13):5775–80. doi: 10.1021/acs.cgd.4c00538 (PMC11232035; doi:10.1021/acs.cgd.4c00538)
Supplement: Supplementary file 1 — cg4c00538_si_001.pdf [file cg4c00538_si_001.pdf]

Unveiling the potential of haloalkenes as electron density acceptors

Juan D. Velasquez[a,$] Noushin Keshtkar,[b $] Víctor Polo,[b,c] Julen Munárriz[b,c] and Jorge Echeverría[a*]

[a]Instituto de Síntesis Química y Catalisis Homogénea (ISQCH) and Departmento de Química Inorgánica, Facultad de Ciencias, Universidad de Zaragoza, Pedro Cerbuna 12, 50009 Zaragoza (Spain).
[b]Departmento de Química Física, Facultad de Ciencias, Universidad de Zaragoza, Pedro Cerbuna 12, 50009 Zaragoza (Spain).
[c]Instituto de Biocomputación y Física de Sistemas Complejos (BIFI), Universidad de Zaragoza, 50009 Zaragoza (Spain)

[$] These authors contributed equally to this work.

SUPPORTING INFORMATION

Scheme S1. Adducts between 2-4 and chloride

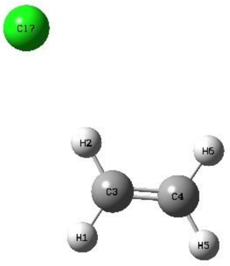

1

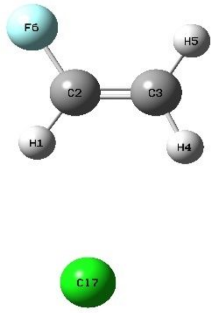

2a

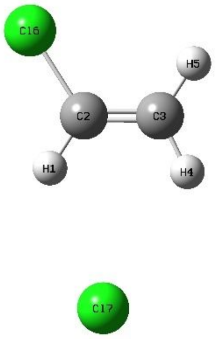

2b

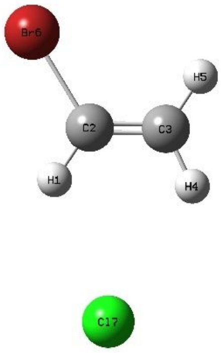

2c

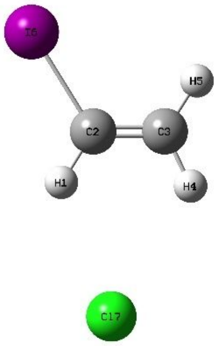

2d

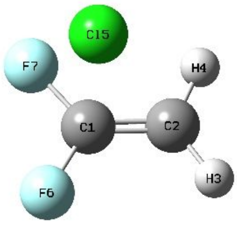

3a

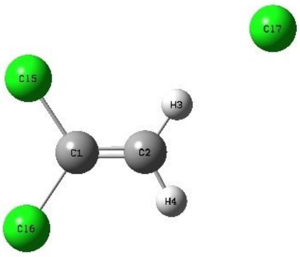

3b

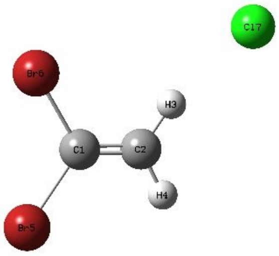

3c

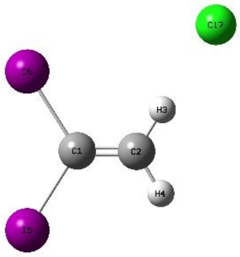

3d

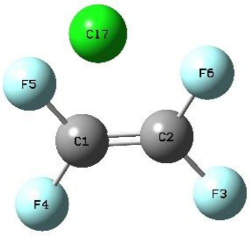

4a

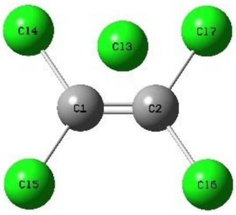

4b

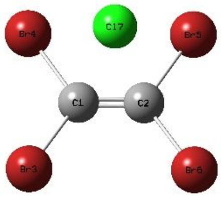

4c

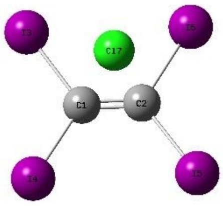

4d

Table S1. NBO results for adducts between 2-4 and chloride.

Alkene	Donor	*Acceptor*	$E^{(2)}$ (kcal/mol)	Charge Transfer
**1**	LP(1)Cl7	BD*(1)H2-C3	0.16	nCl → σ *C-H
	LP(2)Cl7	BD*(1)C3-C4	0.09	nCl → π*C=C
	LP(4)Cl7	BD*(1)H1-C3	0.05	nCl → σ *C-H
	LP(4)Cl7	BD*(1)H2-C3	5.54	nCl → σ *C-H
	LP(4)Cl7	BD*(2)C3-C4	0.11	nCl → π*C=C
**2a**	LP (1)Cl7	BD*(1)H1-C2	0.08	nCl→ σ *C-H
	LP (3)Cl7	BD*(1)H1-C2	0.28	nCl → σ *C-H
	LP (3)Cl7	BD*(1)C3-H4	0.69	nCl → σ *C-H
	LP (3)Cl7	BD*(1)C3-H5	0.10	nCl → σ *C-H
	LP (4)Cl7	BD*(1)H1-C2	2.85	nCl → σ *C-H
	LP (4)Cl7	BD*(1)C2-F6	0.87	nCl → σ *C-F
	LP (4)Cl7	BD*(1)C3-H4	0.44	nCl → σ *C-H
	LP (4)Cl7	BD*(1)C3-H5	0.35	nCl → σ *C-H
**2b**	LP (1)Cl7	BD*(1)H1-C2	0.05	nCl → σ *C-H
	LP (1)Cl7	BD*(1)C2-Cl6	0.08	nCl → σ *C-Cl
	LP (3)Cl7	BD*(1)H1-C2	0.34	nCl → σ *C-H
	LP (3)Cl7	BD*(1)C3-H4	0.90	nCl → σ *C-H
	LP (3)Cl7	BD*(1)C3-H5	0.10	nCl → σ *C-H
	LP (4)Cl7	BD*(1)H1-C2	2.29	nCl → σ *C-H
	LP (4)Cl7	BD*(1)C2-Cl6	1.23	nCl → σ *C-Cl
	LP (4)Cl7	BD*(1)C3-H4	0.74	nCl → σ *C-H
	LP (4)Cl7	BD*(1)C3-H5	0.36	nCl → σ *C-H
**2c**	LP (1)Cl7	BD*(1)C2-Br6	0.11	nCl → σ *C-Br
	LP (3)Cl7	BD*(1)H1-C2	0.32	nCl → σ *C-H
	LP (3)Cl7	BD*(1)C3-H4	1.05	nCl → σ *C-H
	LP (3)Cl7	BD*(1)C3-H5	0.11	nCl → σ *C-H
	LP (4)Cl7	BD*(1)H1-C2	1.89	nCl → σ *C-H
	LP (4)Cl7	BD*(1)C2-Br6	1.56	nCl → σ *C-Br
	LP (4)Cl7	BD*(1)C3-H4	0.84	nCl → σ *C-H
	LP (4)Cl7	BD*(1)C3-H5	0.34	nCl → σ *C-H
**2d**	LP (1)Cl7	BD*(1)C2-I6	0.15	nCl → σ *C-I
	LP (3)Cl7	BD*(1)H1-C2	0.25	nCl → σ *C-H
	LP (3)Cl7	BD*(1)C3-H4	1.12	nCl → σ *C-H
	LP (3)Cl7	BD*(1)C3-H5	0.13	nCl → σ *C-H
	LP (4)Cl7	BD*(1)H1-C2	2.01	nCl → σ *C-H
	LP (4)Cl7	BD*(1)C2-I6	1.90	nCl → σ *C-I
	LP (4)Cl7	BD*(1)C3-H4	0.76	nCl → σ *C-H
	LP (4)Cl7	BD*(1)C3-H5	0.29	nCl → σ *C-H

**3a**	LP(4)Cl5<br>LP(4)Cl5<br>LP(4)Cl5<br>LP(4)Cl5	BD*(1)C1-C2<br>BD*(2)C1-C2<br>BD*(1)C1-F6<br>BD*(1)C1-F7	0.31<br>1.45<br>0.20<br>0.20	nCl → π*C=C<br>nCl → π*C=C<br>nCl → σ *C-F<br>nCl → σ *C-F
**3b**	LP(1)Cl7<br>LP(2)Cl7<br>LP(3)Cl7<br>LP(4)Cl7<br>LP(4)Cl7	BD*(1)C2-H3<br>BD*(1)C1-C2<br>BD*(2)C1-C2<br>BD*(1)C1-C2<br>BD*(1)C2-H3	0.50<br>0.10<br>0.23<br>0.29<br>11.45	nCl → σ *C-H<br>nCl → π*C=C<br>nCl → π*C=C<br>nCl → π*C=C<br>nCl → σ *C-H
**3c**	LP(1)Cl7<br>LP(2)Cl7<br>LP(3)Cl7<br>LP(4)Cl7<br>LP(4)Cl7	BD*(1)C2-H3<br>BD*(1)C1-C2<br>BD*(2)C1-C2<br>BD*(1)C1-C2<br>BD*(1)C2-H3	0.68<br>0.09<br>0.22<br>0.27<br>13.71	nCl → σ *C-H<br>nCl → π*C=C<br>nCl → π*C=C<br>nCl → π*C=C<br>nCl → σ *C-H
**3d**	LP(1)Cl7<br>LP(2)Cl7<br>LP(3)Cl7<br>LP(4)Cl7<br>LP(4)Cl7	BD*(1)C2-H3<br>BD*(2)C1-C2<br>BD*(1)C1-C2<br>BD*(1)C1-C2<br>BD*(1)C2-H3	0.87<br>0.19<br>0.06<br>0.17<br>15.90	nCl → σ *C-H<br>nCl → π*C=C<br>nCl → π*C=C<br>nCl → π*C=C<br>nCl → σ *C-H
**4a**	LP(3)Cl7<br>LP(4)Cl7<br>LP(4)Cl7<br>LP(4)Cl7	BD*(2)C1-C2<br>BD*(2)C1-C2<br>BD*(1)C1-F4<br>BD*(1)C1-F5	0.18<br>1.11<br>0.18<br>0.18	nCl → π*C=C<br>nCl → π*C=C<br>nCl → σ *C-F<br>nCl → σ *C-F
**4b**	LP(3)Cl3<br>LP(3)Cl3	BD*(1)C1-C2<br>BD*(2)C1-C2	0.05<br>1.14	nCl → π*C=C<br>nCl → π*C=C
**4c**	LP(4)Cl7<br>LP(4)Cl7	BD*(1)C1-C2<br>BD*(2)C1-C2	0.08<br>1.23	nCl → π*C=C<br>nCl → π*C=C
**4d**	LP(4)Cl7<br>LP(4)Cl7<br>LP(3)Cl7<br>LP(3)Cl7<br>LP(3)Cl7<br>LP(3)Cl7	BD*(1)C1-C2<br>BD*(2)C1-C2<br>BD*(1)C1-I3<br>BD*(1)C1-I4<br>BD*(1)C1-I5<br>BD*(1)C1-I6	0.10<br>1.26<br>0.07<br>0.07<br>0.07<br>0.07	nCl → π*C=C<br>nCl → π*C=C<br>nCl → σ *C-I<br>nCl → σ *C-I<br>nCl → σ *C-I<br>nCl → σ *C-I

Scheme S2. adducts between 2-4 and acetone.

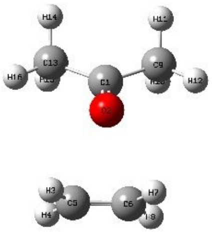

1

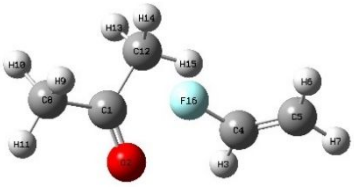

2a

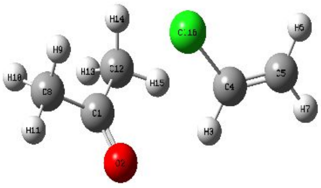

2b

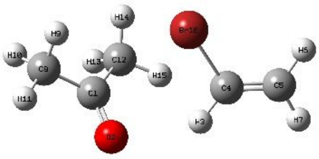

2c

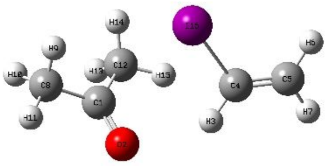

2d

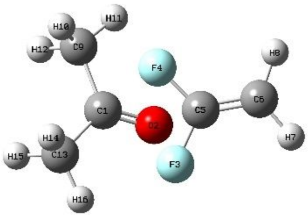

3a

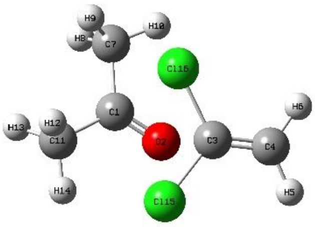

3b

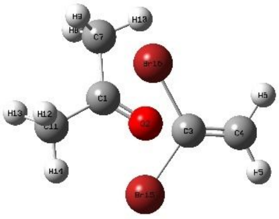

3c

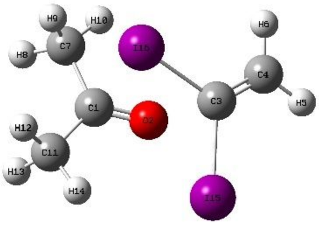

3d

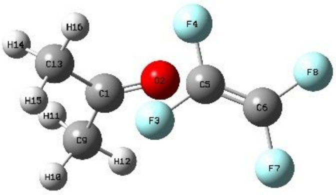

4a

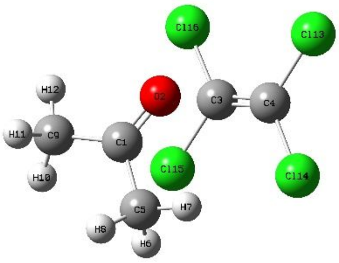

4b

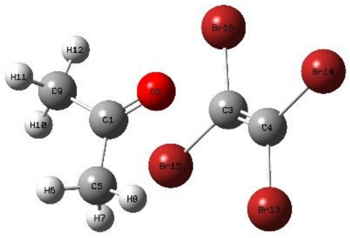

4c

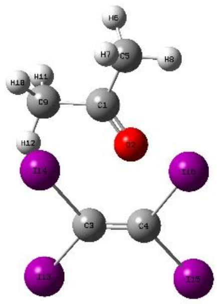

4d

Table S2. NBO results for adducts between 2-4 and acetone.

Alkene	Donor	*Acceptor*	$E^{(2)}$ (kcal/mol)	Charge Transfer
**1**	BD(2)C1-O2	BD*(1)H4-C5	0.10	πC=O→ σ *C-H
	BD(2)C1-O2	BD*(1)C6-H8	0.10	πC=O→ σ *C-H
**2a**	LP(1)O2	BD*(1)H3-C4	0.25	nO → σ *C-H
	LP(1)O2	BD*(1)H3-C4	0.22	nO → σ *C-H
	LP(1)O2	BD*(2)C4-C5	0.06	nO → π*C=C
	BD(2)C1-O2	BD*(1)H3-C4	0.27	πC=O→ σ *C-H
	BD(2)C1-O2	BD*(2)C4-C5	0.13	πC=O→ π*C=C
**2b**	LP(1)O2	BD*(1)H3-C4	0.33	nO → σ *C-H
	LP(2)O2	BD*(1)H3-C4	0.40	nO → σ *C-H
	LP(1)O2	BD*(2)C4-C5	0.08	nO → π*C=C
	BD(2)C1-O2	BD*(1)H3-C4	0.31	πC=O→ σ *C-H
	BD(2)C1-O2	BD*(2)C4-C5	0.15	πC=O→ π*C=C
**2c**	LP(1)O2	BD*(1)H3-C4	0.35	nO → σ *C-H
	LP(1)O2	BD*(1)C4-C5	0.10	nO → π*C=C
	LP(2)O2	BD*(1)H3-C4	0.45	nO → σ *C-H
	BD(2)C1-O2	BD*(1)H3-C4	0.30	πC=O→ σ *C-H
	BD(2)C1-O2	BD*(1)C4-C5	0.15	πC=O→ π*C=C
**2d**	LP(1)O2	BD*(1)H3-C4	0.32	nO → σ *C-H
	LP(1)O2	BD*(1)C4-C5	0.11	nO → π*C=C
	LP(2)O2	BD*(1)H3-C4	0.48	nO → σ *C-H
	LP(2)O2	BD*(2)C4-C5	0.05	nO → π*C=C
	BD(2)C1-O2	BD*(1)H3-C4	0.26	πC=O→ σ *C-H
	BD(2)C1-O2	BD*(1)C4-C5	0.15	πC=O→ π*C=C
**3a**	LP(1)O2	BD*(1)C5-C6	0.05	nO → π*C=C
	LP(1)O2	BD*(2)C5-C6	0.08	nO → π*C=C
	BD(2)C1-O2	BD*(2)C5-C6	0.40	πC=O→ π*C=C
**3b**	LP(1)O2	BD*(2)C3-C4	0.07	nO → π*C=C
	BD(2)C1-O2	BD*(2)C3-C4	0.36	πC=O→ π*C=C
**3c**	LP(1)O2	BD*(2)C3-C4	0.08	nO → π*C=C
	LP(2)O2	BD*(2)C3-C4	0.06	nO → π*C=C
	BD(2)C1-O2	BD*(2)C3-C4	0.37	πC=O→ π*C=C
**3d**	LP(1)O2	BD*(2)C3-C4	0.15	nO → π*C=C
	LP(2)O2	BD*(2)C3-C4	0.16	nO → π*C=C
	BD(1)C1-O2	BD*(2)C3-C4	0.05	πC=O→ π*C=C
	BD(2)C1-O2	BD*(2)C3-C4	0.32	πC=O→ π*C=C
**4a**	LP(1)O2	BD*(2)C5-C6	0.19	nO → π*C=C
	LP(2)O2	BD*(2)C5-C6	0.42	nO → π*C=C
	BD(2)C1-O2			

		BD*(2)C5-C6	0.12	πC=O→ π*C=C
**4b**	LP(1)O2<br>LP(2)O2	BD*(2)C3-C4<br>BD*(2)C3-C4	0.06<br>0.38	nO → π*C=C<br>nO → π*C=C
**4c**	LP(1)O2<br>LP(2)O2	BD*(2)C3-C4<br>BD*(2)C3-C4	0.12<br>0.40	nO → π*C=C<br>nO → π*C=C
**4d**	LP(1)O2<br>LP(2)O2	BD*(2)C3-C4<br>BD*(2)C3-C4	0.15<br>0.37	nO → π*C=C<br>nO → π*C=C

Table S3. Cartesian coordinates of optimized systems.

Adduct 1 and chloride / M06-2X

H	-1.95892	-1.58718	-0.00003
H	-0.54979	-0.37474	0.00006
C	-1.62536	-0.55367	0.00001
C	-2.51055	0.43200	-0.00001
H	-3.58050	0.24990	-0.00005
H	-2.18860	1.46703	0.00004
Cl	1.94666	0.05736	-0.00000

Adduct 2a and chloride / M06-2X

H	-0.34033	0.79814	-0.00008
C	-1.18432	0.11778	-0.00003
C	-1.08162	-1.19268	-0.00000
H	-0.07671	-1.59371	-0.00004
H	-1.95435	-1.83285	0.00006
F	-2.39610	0.73314	0.00002
Cl	2.20776	0.14586	0.00001

Adduct 2b and chloride / M06-2X

H	-0.10350	-0.64314	0.00035
C	0.65620	0.12991	0.00019
C	0.36305	1.41307	-0.00003
H	-0.69329	1.66015	0.00013
H	1.12388	2.18322	-0.00018
Cl	2.30872	-0.45259	-0.00004
Cl	-2.68770	-0.28024	-0.00003

Adduct 2c and chloride / M06-2X

H	-0.76243	-0.56704	0.00004
C	-0.06846	0.26506	0.00003
C	-0.45233	1.52283	-0.00000

H	-1.52581	1.68790	0.00001
H	0.24523	2.35036	-0.00003
Br	1.78086	-0.23295	-0.00000
Cl	-3.36249	-0.35562	-0.00000

Adduct 2d and chloride / M06-2X

H	-1.25244	-0.50635	-0.00010
C	-0.57541	0.34119	-0.00005
C	-0.99407	1.58849	0.00001
H	-2.07304	1.72246	-0.00003
H	-0.32698	2.44100	0.00006
I	1.47212	-0.15622	0.00000
Cl	-3.82077	-0.40915	0.00001

Adduct 3a and chloride / M06-2X

C	-1.10003	-0.03279	-0.00018
C	-1.30683	-1.32981	0.00047
H	-1.31979	-1.85760	0.93845
H	-1.31783	-1.85869	-0.93693
Cl	2.09536	-0.06200	0.00043
F	-1.03116	0.71992	1.07348
F	-1.02911	0.71851	-1.07465

Adduct 3b and chloride / M06-2X

C	-0.88544	-0.16106	-0.00008
C	0.22251	-0.87004	-0.00025
H	1.22230	-0.42279	-0.00027
H	0.14738	-1.94941	-0.00030
Cl	-0.92128	1.56536	-0.00002
Cl	-2.47625	-0.87573	0.00010
Cl	3.55093	-0.18617	0.00007

Adduct 3c and chloride / M06-2X

C	0.27281	0.31078	0.00056
C	-0.71501	1.17834	0.00113
H	-1.77213	0.87929	0.00131
H	-0.48412	2.23649	0.00141
Br	2.10817	0.83473	-0.00023
Br	0.00652	-1.55892	0.00003
Cl	-4.06499	0.78212	-0.00035

Adduct 3d and chloride / M06-2X

C	0.08825	0.51388	-0.00008
C	-0.60523	1.63292	-0.00011
H	-1.70668	1.62900	-0.00010
H	-0.08717	2.58534	-0.00011
I	2.19638	0.50918	0.00002
I	-0.83276	-1.35800	-0.00000
Cl	-3.96332	1.64073	0.00004

Adduct 4a and chloride / M06-2X

C	-0.56287	0.77858	0.00025
C	-1.05169	-0.43878	-0.00002
F	-1.29617	-1.12667	-1.09573
F	-0.31651	1.45685	-1.09420
F	-0.31529	1.45582	1.09497
F	-1.29487	-1.12712	1.09583
Cl	2.27605	-0.46874	-0.00054

Adduct 4b and chloride / M06-2X

C	-0.66494	0.00863	-0.57229
C	0.66411	0.00864	-0.57306
Cl	0.00191	-0.03943	2.59977
Cl	-1.57731	1.45050	-0.52644
Cl	-1.57727	-1.43388	-0.56948
Cl	1.57649	-1.43383	-0.57139
Cl	1.57647	1.45055	-0.52823

Adduct 4c and chloride / M06-2X

C	-0.66359	0.00025	-0.35282
C	0.66376	0.00036	-0.35322
Br	-1.67631	-1.57518	-0.31322
Br	-1.67662	1.57534	-0.31174
Br	1.67582	1.57614	-0.31188
Br	1.67677	-1.57479	-0.31364
Cl	0.00065	-0.00334	2.82371

Adduct 4d and chloride / M06-2X

C	0.66287	-0.00013	-0.28888
C	-0.66277	-0.00036	-0.28899
I	1.81089	-1.73661	-0.21622
I	1.80962	1.73734	-0.21688
I	-1.81074	1.73608	-0.21730
I	-1.80953	-1.73772	-0.21624
Cl	-0.00080	0.00299	2.90583

Adduct 1 and acetone / M06-2X

C	-1.05244	0.00072	0.20963
O	-0.81525	0.00131	1.39157
H	1.56568	-1.22031	0.65380
H	2.76839	-1.23254	-0.75339
C	2.16744	-0.66300	-0.05459
C	2.17024	0.66048	-0.05356
H	1.57106	1.21931	0.65583
H	2.77366	1.22850	-0.75148
C	-1.20151	1.28096	-0.57419
H	-0.44835	1.30790	-1.36471
H	-2.18110	1.31388	-1.05525
H	-1.08015	2.13821	0.08293
C	-1.20472	-1.28030	-0.57228
H	-2.18317	-1.31016	-1.05586
H	-0.44972	-1.31146	-1.36090
H	-1.08841	-2.13704	0.08643

Adduct 2a and acetone / M06-2X

C	-1.44565	0.26191	-0.18075
O	-1.11885	0.58692	-1.29604
H	1.27015	0.41006	-1.12725
C	1.83334	-0.05199	-0.32705
C	3.07003	0.21239	0.03238
H	3.54124	-0.30731	0.85467
H	3.62547	0.96227	-0.50979
C	-2.23362	-0.99521	0.08238
H	-1.58204	-1.70761	0.59361
H	-3.07649	-0.79090	0.74454
H	-2.57968	-1.42473	-0.85379
C	-1.08133	1.09066	1.02578
H	-1.98510	1.56449	1.41655
H	-0.67614	0.45577	1.81448
H	-0.36164	1.85798	0.75071
F	1.10205	-0.98021	0.32091

Adduct 2b and acetone / M06-2X

C	1.69769	0.38390	0.11735
O	1.32682	0.96033	1.11085
H	-0.99195	0.91841	0.94783
C	-1.72898	0.39789	0.35154
C	-2.88996	0.89388	-0.02692
H	-3.58830	0.32970	-0.62953
H	-3.15915	1.89686	0.27584
C	2.55492	-0.85321	0.19000
H	1.96347	-1.70441	-0.15527
H	3.41467	-0.76338	-0.47612
H	2.88080	-1.02564	1.21205
C	1.33095	0.86669	-1.26384

H	2.21793	1.30315	-1.73010
H	1.00717	0.03297	-1.88764
H	0.54841	1.61939	-1.20389
Cl	-1.21737	-1.20144	-0.08522

Adduct 2c and acetone / M06-2X

C	2.08370	0.39725	0.10571
O	1.77669	1.06026	1.06663
H	-0.50064	1.36617	0.90805
C	-1.34069	0.97005	0.35453
C	-2.39708	1.65554	-0.03171
H	-3.20818	1.20972	-0.59071
H	-2.46314	2.70652	0.22053
C	2.79404	-0.92392	0.24627
H	2.11224	-1.71767	-0.06808
H	3.66351	-0.96267	-0.41223
H	3.09021	-1.08436	1.27925
C	1.78279	0.85196	-1.30075
H	2.71341	1.18860	-1.76500
H	1.39569	0.02621	-1.89831
H	1.07094	1.67406	-1.28623
Br	-1.13211	-0.87411	-0.03328

Adduct 2d and acetone / M06-2X

C	2.42556	0.30314	0.10300
O	2.17756	0.99633	1.05968
H	-0.02899	1.63323	0.89870
C	-0.93230	1.38512	0.35787
C	-1.84096	2.25353	-0.03903
H	-2.72925	1.96673	-0.58541
H	-1.70354	3.30437	0.18938
C	3.01028	-1.07726	0.25258
H	2.25819	-1.80619	-0.05912
H	3.87339	-1.20016	-0.40402
H	3.28829	-1.25876	1.28711
C	2.17495	0.77771	-1.30687
H	3.13383	1.04785	-1.75725
H	1.73870	-0.01777	-1.91161
H	1.52385	1.64899	-1.30089
I	-1.09057	-0.66306	-0.01962

Adduct 3a and acetone / M06-2X

C	1.53284	-0.02562	-0.37924
O	0.85963	-0.18502	-1.36683
F	-1.43215	1.25809	0.09501
F	-0.88521	-0.55853	1.09901
C	-1.67810	-0.03022	0.18379

C	-2.57444	-0.68868	-0.50878
H	-3.16953	-0.16411	-1.23712
H	-2.68884	-1.74850	-0.35572
C	2.20920	-1.18103	0.31650
H	1.93088	-1.19334	1.37174
H	1.92727	-2.11775	-0.15658
H	3.29282	-1.05325	0.26875
C	1.73394	1.33720	0.23588
H	1.25910	1.35579	1.21955
H	2.79718	1.53372	0.38448
H	1.28969	2.10168	-0.39549

Adduct 3b and acetone / M06-2X

C	-1.91206	0.26879	0.38881
O	-1.27898	0.37120	1.40966
C	1.49582	-0.30757	0.21131
C	1.96275	-0.75610	1.35827
H	2.44993	-0.08066	2.04547
H	1.84407	-1.79571	1.62443
C	-2.71448	-0.96984	0.07203
H	-3.77784	-0.73535	0.16517
H	-2.54043	-1.28612	-0.95735
H	-2.45705	-1.76821	0.76302
C	-1.94895	1.37044	-0.64098
H	-1.39142	1.04242	-1.52219
H	-2.97506	1.56517	-0.95776
H	-1.49240	2.27226	-0.24209
Cl	1.62411	1.33374	-0.28295
Cl	0.68609	-1.32301	-0.92485

Adduct 3c and acetone / M06-2X

C	2.20233	-0.95045	0.30247
O	1.58291	-1.14268	1.31875
C	-0.99241	0.47037	0.62931
C	-1.18651	0.69486	1.91203
H	-1.75651	-0.00400	2.50768
H	-0.76282	1.56856	2.38650
C	3.30108	0.08160	0.22456
H	4.26480	-0.43323	0.19461
H	3.21534	0.66612	-0.69231
H	3.26505	0.73199	1.09459
C	1.92630	-1.72967	-0.95927
H	1.43616	-1.06528	-1.67617
H	2.85842	-2.07004	-1.41347
H	1.27184	-2.57058	-0.74594
Br	-1.67699	-1.04216	-0.25690
Br	0.02098	1.63980	-0.45339

Adduct 3d and acetone / M06-2X

C	-2.02116	1.91795	0.14964
O	-1.19760	2.04338	1.02118
C	0.57757	-0.53738	0.86657
C	0.56096	-0.57456	2.18458
H	1.30358	-0.03893	2.76163
H	-0.20401	-1.12477	2.71684
C	-3.44901	1.53620	0.45411
H	-4.13921	2.23643	-0.02012
H	-3.64843	0.54824	0.03276
H	-3.61037	1.51493	1.52855
C	-1.68359	2.14370	-1.30367
H	-2.07056	1.32927	-1.91770
H	-2.16614	3.06502	-1.63987
H	-0.60684	2.22963	-1.42735
I	2.01600	0.53189	-0.19312
I	-0.86856	-1.53231	-0.26559

Adduct 4a and acetone / M06-2X

C	-2.21104	-0.23094	-0.32245
O	-1.18765	-0.46078	-0.91814
F	0.34889	0.60549	1.32097
F	0.99258	1.66889	-0.48063
C	1.14667	0.61152	0.27690
C	2.01157	-0.34612	0.02701
F	2.15623	-1.40481	0.78969
F	2.80985	-0.34789	-1.01378
C	-3.21188	-1.31745	-0.01759
H	-3.44013	-1.32947	1.04972
H	-4.14633	-1.10745	-0.54244
H	-2.82355	-2.28273	-0.33032
C	-2.55491	1.15617	0.16000
H	-3.56303	1.42928	-0.15619
H	-2.54408	1.16771	1.25220
H	-1.83213	1.87468	-0.21736

Adduct 4b and acetone / M06-2X

C	-2.27289	-0.21947	0.85892
O	-1.25784	-0.32085	1.50268
C	0.76448	0.68625	-0.48618
C	1.21464	-0.51991	-0.14380
C	-2.87451	-1.39651	0.13312
H	-2.75992	-1.23966	-0.94243
H	-2.36586	-2.31301	0.42028
H	-3.94389	-1.46853	0.33916
C	-3.00903	1.09291	0.74450
H	-3.33844	1.26637	-0.28041

H	-3.90311	1.04758	1.37173
H	-2.37305	1.90730	1.08292
Cl	2.36051	-0.74547	1.09715
Cl	0.65085	-1.94705	-0.89494
Cl	-0.42276	0.89999	-1.70191
Cl	1.28262	2.11648	0.28488

Adduct 4c and acetone / M06-2X

C	2.08761	0.56350	1.83996
O	0.91572	0.45464	2.10397
C	-0.10323	-0.64590	-0.54005
C	-0.88697	0.39220	-0.26021
C	2.69850	1.89129	1.46890
H	3.62196	2.05857	2.02608
H	2.95717	1.87007	0.40711
H	1.98894	2.69416	1.65114
C	3.01873	-0.62378	1.87303
H	3.70786	-0.60365	1.02853
H	3.61697	-0.57004	2.78647
H	2.44435	-1.54739	1.87394
Br	-0.45869	2.14716	-0.76602
Br	-2.47590	0.22643	0.71351
Br	1.51704	-0.46582	-1.47588
Br	-0.48390	-2.39356	0.01712

Adduct 4d and acetone / M06-2X

C	-1.41435	-0.83494	2.58928
O	-0.30412	-0.37568	2.48334
C	-0.32037	0.48546	-0.49127
C	0.79635	-0.21354	-0.31763
C	-1.66226	-2.32239	2.59307
H	-2.32829	-2.59797	3.41277
H	-2.16325	-2.59377	1.65996
H	-0.72010	-2.85954	2.66579
C	-2.62574	0.05340	2.73844
H	-3.47281	-0.33776	2.17481
H	-2.91125	0.07184	3.79375
H	-2.38738	1.06464	2.41541
I	-0.46066	2.54112	-0.16764
I	-2.14156	-0.37972	-1.04556
I	2.60013	0.63735	0.28494
I	0.90348	-2.28459	-0.55590
